# A protocol for single molecule imaging and tracking of processive myosin motors

**DOI:** 10.1016/j.mex.2019.08.011

**Published:** 2019-08-23

**Authors:** Lucia Gardini, Claudia Arbore, Marco Capitanio, Francesco Saverio Pavone

**Affiliations:** a LENS - European Laboratory for Non-Linear Spectroscopy, Via Nello Carrara 1, 50019, Sesto Fiorentino, Italy; b National Institute of Optics–National Research Council, Largo Fermi 6, 50125, Florence, Italy; c Department of Physics and Astronomy, University of Florence, Via Sansone 1, 50019, Sesto Fiorentino, Italy

**Keywords:** Single molecule motility assay, Total internal reflection fluorescence (TIRF) microscopy, Single molecule biophysics, Single particle tracking, Quantum dots, Myosin-5B

## Abstract

Myosin is a large family of actin-based molecular motors, which includes efficient intracellular transporters that move cargoes and material essential for cell’s life. Here, we describe protocols for labelling single myosin motors with quantum dots, tracking them in an in vitro reconstituted single-molecule motility assay, acquiring image stacks and analyzing them. We describe the required steps to obtain trajectories of single myosin motors from which fundamental biophysical parameters such as the motor velocity, run length and step size can be derived. We also describe protocols for an ensemble actin gliding assay, which is valuable to test the motor viability and its ensemble properties. The protocols allow probing the effect of changes in nucleotides, ions, and buffer composition on the motor properties and are easily generalizable to track the movements of different motor proteins.

## Graphical abstract

**Figure:**
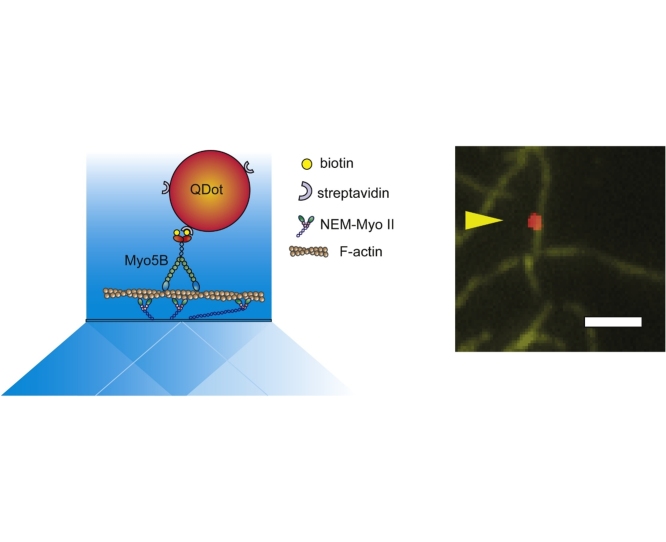

Specifications Table

**Table:** 

Subject Area:	Biochemistry, Genetics and Molecular Biology
More specific subject area:	Biophysics
Protocol name:	Single molecule motility assay
Reagents/tools:	All materials (reagents and equipment) are listed in the appropriate article section for clarity
Experimental design:	We describe the labelling of a recombinant myosin-5B with a quantum dot, the polymerization and labelling of actin filaments and the assembly of an in vitro single molecule motility assay to track myosin movements on actin under total internal fluorescence microscopy (TIRFM). We also describe an ensemble gliding assay in which multiple myosin-5B molecules translocate actin filaments and are visualized under fluorescence microscopy.
Trial registration:	NA
Ethics:	NA

Value of the Protocol:

**Table:** 

• Single molecule techniques allow tracking the movements of molecular motors with nanometer accuracy to investigate their mechanochemical and biophysical properties [1,2,3]. Here we share a protocol for QD-labelling purified processive myosin motors and observe them while they move on actin filaments immobilized on a coverslip surface. • The protocol allows extracting motor trajectories using our freely available tracking software (PROOF, available as supplementary material in Gardini et al. [4]) or different tracking methods [5,6] with sufficient resolution to derive fundamental biophysical parameters such as the motor velocity, run length and step size and how they vary with nucleotides, ions, and buffer composition [7]. Such data is fundamental to dissect the molecular mechanisms at the base of motor protein function and test different chemo-mechanical models [8,9]. • We also share a protocol for an ensemble actin gliding assay, which is valuable to test the motor viability and its ensemble properties.

## Description of protocol

Myosin is a family of actin-based motor proteins that hydrolyze ATP to generate force and movement and perform a broad range of functions, such as muscle contraction, hearing, vision, and cell motility [10]. Among these, several myosins transport intracellular cargoes for long distances inside living cells. For this reason, such motors are named *processive*, which means that they perform a large number of steps and ATP hydrolysis cycles before dissociating from actin [9]. Single molecule techniques allow direct observation of biophysical properties of motor proteins that would not be otherwise accessible. Here, we describe protocols for *in vitro* characterization of processive myosin motors at the single molecule level. We describe a single molecule motility assay in which single myosin molecules are labeled with single quantum dots and move along fluorescent actin filaments attached to the surface of a glass coverslip. In this configuration it is possible to measure fundamental biophysical properties of the motor such as the step size, run length, and velocity under varying nucleotides, Ca^2+^, and buffer composition. We also describe an *in vitro* gliding assay in which myosins attached onto a glass coverslip translocate fluorescent actin filaments in the presence of ATP in solution. By measuring the average gliding velocity of the filaments, it is possible to test protein viability and processivity of motor ensembles.

We describe protocols that were optimized to study the biophysical properties of myosin-5B [7], but can be easily generalized to the study of other myosin motors.

### Myosin-5B expression and biotinylation

Recombinant myosin-5B heavy meromyosin (HMM) was expressed in Sf9 cells using the Baculovirus expression system. Description of protocols for the Baculovirus expression system is beyond the scope of the present article and protocols were performed as recommended by the manufacturer (Thermo Fisher Scientific). Protein purification was performed following standard techniques, whose description is also beyond the scope of the present article. In this section, we briefly describe the strategy that we adopted to engineer a recombinant myosin with a tag for the specific binding in the single molecule and gliding assays. The C-terminus of myosin-5B heavy chain was biotinylated using the Avi-tag-BirA system [11]. The biotin tag was used for the specific binding of myosin to either streptavidinated QDs in the single molecule motility assay (Section 5) or to a streptavidinated coverslip in the gliding assay (Section 4).To this end, a cDNA construct encoding for amino acids 1–1095 of murine myosin-5B heavy meromyosin (HMM) (Accession number NM_201600.2, MW 127 kDa) with a C-terminal Avi-tag was inserted in a modified pFastBac1 vector encoding a C-terminal Flag-tag with standard cloning techniques. After recombinant baculovirus generation, Sf9 insect cells from ThermoScientific (B82501) were infected with recombinant baculoviruses encoding Avi-tag myosin-5B HMM and calmodulin (CaM). Avi-tag myosin-5B HMM was purified via Flag capture [12] followed by ion exchange chromatography through Q-Sepharose Fast Flow resin (GE Healthcare Life Sciences).

**Fig. 1:**
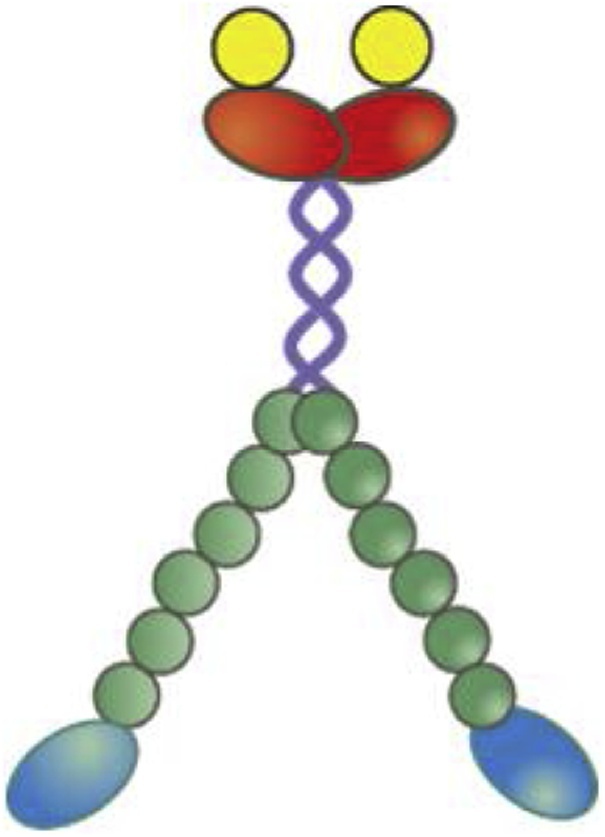
Sketch of the biotinylated myosin (bio-HMM-5B) structure. The picture shows the two myosin heads (the motor units, blue), the six calmodulin molecules bound to the light-chain binding domains (green), the dimerization domain (purple), and the C-terminus (red) with biotin tags (yellow).

Myosin-5B was biotinylated on the Flag-resin through incubation with BirA biotin ligase and d-biotin (Avidity). We loaded 1 ml of solution containing 20 mM ATP, 20 mM MgOAc, 100 μM d-Biotin and 150 μg BirA enzyme in a 2 ml Flag resin while the protein was trapped in the resin and incubated it for 90 min at 4 °C. The protein, named herein bio-HMM-5B, was then eluted from the column following the standard Flag-resin elution protocol. Since two heavy chains dimerize to form the active myosin-5B motor, we end up with a molecule with two biotin tag at the distal end of the motor unit, as represented in Fig. 1.

### F-actin polymerization and labelling

F-actin was polymerized from purified G-actin and labelled with rhodamine-phalloidin for visualization in both the single molecule motility and gliding assays.

Materials:

• G-actin protein (Cytoskeleton, AKL99 1 mg), reconstituted to 10 mg/ml from powder following manufacturer instructions, final buffer 5 mM Tris−HCl pH 8.0, 0.2 mM CaCl_2_, 0.2 mM ATP, 5% (w/v) sucrose and 1% (w/v) dextran
• Actin polymerization buffer 10 X, (Cytoskeleton, BSA02: 100 mM Tris HCl, 20 mM MgCl2, 500 mM KCl, 10 mM ATP, 50 mM guanidine carbonate, pH 7.5)
• DL-Dithiothreitol (DTT) 1 M in ultrapure water
• Rhodamine Phalloidin (Phalloidin–Tetramethylrhodamine B isothiocyanate) 250 μM in methanol (Sigma P1951). (Tip: aliquotes can be stored at −20 °C for several months)
• MilliQ ultrapure water

Methods:

1 In a 0.5 ml tube mix 69 μl ultrapure water, 10 μl actin polymerization buffer 10X, 20 μl G-actin 10 mg/ml and 1 μl DTT 1 M. Mix gently without pipetting
2 Put on ice for > 1 hour
3 In a new 0.5 ml tube take 25 μl polymerized F-actin and add 19.5 μl ultrapure water, 2.5 μl actin polymerization buffer 10X, 1 μl DTT 1 M and 2 μl rhodamine phalloidin 250 μM
4 Leave on ice overnight. (Tip: labelled F-actin can be stored on ice for about a week. Prior dilution at experimental concentration mix it gently without pipetting to disperse it)

### Flow chamber preparation

**Fig. 2:**
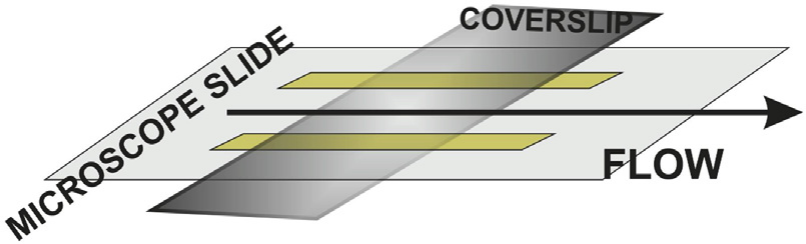
Sketch of the flow chamber. A glass coverslip, smeared with nitrocellulose, is attached onto a microscope slide through double sticky tape stripes to form a flow-cell of about 20 μl volume. Solutions are flown from one side of the chamber with a pipette and sucked from the other side through a filter paper to create a flow in the arrow direction.

In both the single molecule motility and gliding assays, we assembled the *in vitro* system inside a small-volume flow chamber in which the coverslip was covered with nitrocellulose for protein adhesion (Fig. 2). The glass coverslip in the chamber represented in Fig. 2 is placed on the top, as during sample preparation, when solutions are fluxed in the chamber as described in the next sections. Instead, imaging was performed on an inverted microscope (see section 6) in which the coverslip faced downwards.

Materials:

• Pure Ethanol
• Nitrocellulose 1%: 10 mg nitrocellulose 0.45 μm pore size (Sigma N8267) dissolved in 1 ml pentyl acetate solution. (Tip: nitrocellulose 1% can be stored at 4 °C for few months)
• Double sticky tape (˜ 60 μm thick)
• Glass coverslips: 24 x 24 mm and 60 x 24 mm, ˜ 150 μm thick. Glass slides: 26 x 76 mm, ˜ 1 mm thick (purchased from VWR).

Methods:

1 Take a glass coverslip (24 x 24 mm) and cleanse it carefully with paper soaked with pure ethanol. Then rinse it directly with pure ethanol, by handling it carefully with clean tweezers. Dry it under a gentle flow of nitrogen. No visible residues must be left on the glass surface. If perfect cleaning is not reached after a first cleaning, repeat the cleaning procedure a second time
2 Smear 2 μl of nitrocellulose solution on one surface of the coverslip by means of a second clean coverslip (24 x 60 mm), and wait for it to be completely dry (see video article in ref [13])
3 Take a microscope slide (26 x 76 mm) and clean it carefully with paper soaked with pure ethanol. Dry it under nitrogen flow to get rid of coarse residues.
4 Cut two narrow stripes of double sticky tape (˜ 3 mm large) and stick them on one side of the microscope slide in order to create a chamber of about 20 μl final volume, as shown in Fig. 2. Final volume of the chamber can be varied by either adjusting the distance between the tape stripes or choosing tape with different thickness.
5 By handling the coverslip (prepared at step 2) with clean tweezers, close the chamber with the nitrocellulose layer facing the inside of the chamber.

### Gliding assay

**Fig. 3:**
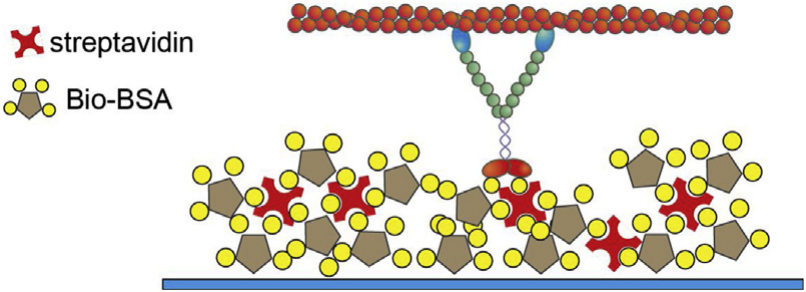
Sketch of the gliding assay. Successive layers of nitrocellulose (blue), biotinylated BSA (BSA, brown; biotin, yellow), and streptavidin (red) are adhered on a coverslip surface. Biotinilated myosin bio-HMM-5B is then attached on top of the streptavidin layer. The surface is passivated through a final layer of biotinylated BSA.

In the gliding assay, bio-HMM-5B is attached to the coverslip surface in the flow chamber as shown in Fig. 3 and rhodamine-labeled F-actin is fluxed inside the chamber and allowed to bind to myosin in the absence of ATP. As we flux ATP into the chamber, we observe actin filaments smoothly translocating on the coverslip surface.

Materials:

• Flow chamber (Section 3)
• Motility Buffer with KCl (MB-KCl): 20 mM MOPS pH 7.4, 1 mM MgCl_2_, 0.1 mM EGTA, 50 mM KCl, and 1 mM DTT. (Tip: filter sterilize and store it at 4 °C for several weeks)
• Biotinylated-BSA 1 mg/ml in MB-KCl (Sigma, A8549). (Tip: store Bio-BSA in 10 mg/ml aliquotes at −80 °C for several months and dilute it at 1 mg/ml before starting the experiments)
• Streptavidin 1 mg/ml in MB-KCl (ThermoFisher, 21,145). (Tip: store Streptavidin 5 mg/ml aliquots at −80 °C for several months and dilute it at 1 mg/ml before starting the experiments)
• Imaging Buffer (IB): MB-KCl buffer supplemented with 1.2 μM glucose oxidase (Sigma G7141), 0.2 μM catalase (Sigma C40), 4.2 μM alpha-casein (Sigma, C6780), 17 mM glucose, and 20 mM DTT. (Tip: glucose oxidase and catalase can be aliquoted at 5 mg/ml and stored at -80 for several months; alpha-casein can be stored in 10 mg/ml aliquotes at −80 °C for several months; glucose can be stored at 250 mg/ml at 4 °C for several months. Owing to deterioration of the enzymes, IB must be prepared fresh every ˜ 5 h)
• Reaction Mix (RM): IB supplemented with 2 μM calmodulin (CaM) (Merk, 208,783) and the desired ATP concentration (maximum velocity is measured at saturating [ATP] = 1–2 mM). (Optional): ATP regenerating system (2 mM creatine phosphate (Sigma 27,920), 100 μg/ml creatine phosphokinase (Sigma C3755), prepare them fresh). ATP regeneration system is very important at low ATP concentration (< 500 μM) to restore ATP from ADP + Pi in the solution and maximize the observation time. (Tip: calmodulin can be stored in aliquotes at 200 μM at −80 °C for several months, while ATP can be stored at 1 mM at −20 °C for several months.)
• Bio-HMM-5B (Section 1)
• Rhodamine-phalloidin labeled F-actin (Section 2)

Methods:

1 Prepare a flow cell as described in Section 3.
2 Flux biotinylated BSA 1 mg/ml and incubate for 3 min.
3 Wash with 3 volumes of MB-KCl.
4 Flux streptavidin 1 mg/ml and incubate for 5 min.
5 Wash with 4 volumes of MB-KCl.
6 Flux bio-HMM-5B 0.4 μM and incubate for exactly 5 min.
7 While incubating, prepare IB and RM.
8 Wash with 4 volumes of biotinylated BSA 1 mg/ml.
9 Flux 23 nM rhodamine phalloidin F-Actin (in MB-KCl supplemented with 10 mM DTT), incubate for 2 min.
10 Wash with 4 volumes of IB.
11 (Optional) Check under the microscope that a reasonable number of actin filaments are firmly bound to the coverslip surface.
12 Wash with RM with the desired ATP concentration.
13 (Optional) Close carefully the chamber with silicone grease. Alternatively, leave the flow cell open to change the actin and ATP concentration on the same sample.
14 Acquire images.

### Single molecule motility assay

**Fig. 4:**
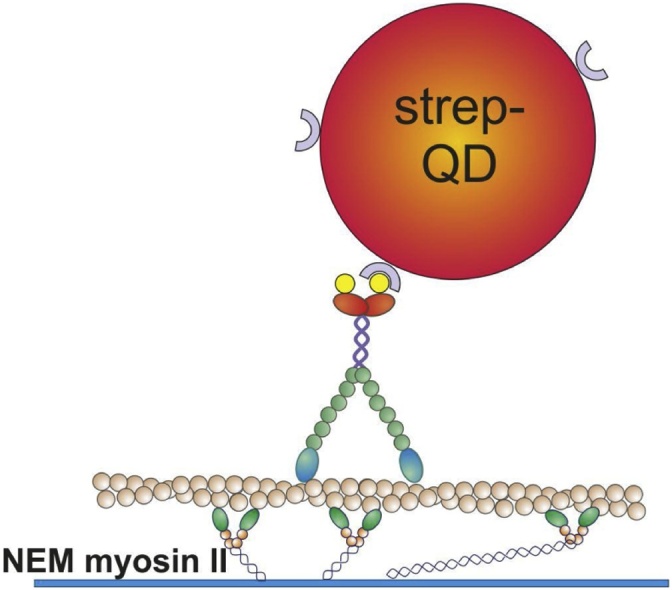
Sketch of the single molecule motility assay. Fluorescently-labelled actin filaments are attached on the nitrocellulose-coated coverslip surface through a layer of inactivated NEM-myosin II. Single biotinylated myosin-5B molecules attached to a streptavidinated QD walk along actin in the presence of ATP.

In the single molecule motility assay, bio-HMM-5B is first attached to streptavidinated QDs at single molecule concentration. Then, a flow chamber is built in which fluorescently-labelled actin filaments are attached on a bed of inactivated N-ethylmaleimide (NEM) myosin II on the coverslip surface [14]. QD-labelled HMM-5B (QD-HMM-5B) are then fluxed into the chamber together with ATP and the movements of single QDs translocating along actin are recorded. In our configuration, HMM-5B is bound to a single streptavidinated Quantum Dot through a biotinylation domain placed at the C-terminus, in substitution of the cargo-binding site (Section 1). Therefore, position of the fluorophore reflects movements of the center of mass of the protein, differently from other labelling geometries [15,16], and interactions of the Quantum Dot with the glass surface and with the N-terminus motor domain are minimized (Fig. 4).

#### Labeling of quantum dots with a single myosin motor

Materials:

• Bio-HMM-5B 0.04-0.2 μM
• Streptavidin-QD 655 nm (Life Technologies Q10123MP) 0.2 μM
• Myosin-V Buffer (VB): 0.5 M NaCl, 10 mM MOPS, 0.1 mM EGTA, 3 mM NaN_3_, pH 7.3. (Tip: filter sterilize and store at 4 °C for several weeks)
• D-Biotin 10 mg/ml in VB. (Tip: store aliquotes at 10 mg/ml at −80 °C and thaw right before experiments)
• Binding Buffer (BB): VB supplemented with 0.1 mg/ml α-casein, 2 μM CaM, 3 mM DTT.

Methods

1 In a 0,5 ml tube, mix1 μl of bio-HMM-5B 0.04-0.2 μM, 1 μl of streptavidin-QDs 0.2 μM, and 2.5 μl of BB. The probability of having no more than one myosin head attached per QD increases as the bio-HMM-5B concentration decreases and, assuming Poisson statistics, it is about 91% at 1:5 bio-HMM-5B:QD molar ratio [17].
2 Put on ice for > 30 min.
3 10 min before fluxing in the chamber, add 0.5 μl of D-Biotin 10 mg/ml to saturate all free streptavidin sites on the QDs. Leave all components on ice during assembly of the single molecule motility assay (next section).

#### Assembly of the single molecule assay

Materials:

• Flow chamber (Section 3)
• Motility Buffer with KCl (MB-KCl): 20 mM MOPS pH 7.4, 1 mM MgCl2, 0.1 mM EGTA, 50 mM KCl, and 1 mM DTT. (Tip: filter sterilize and store it at 4 °C for a couple of weeks)
• Motility Buffer with α-casein (MBα): MB-KCl supplemented with 0.1 mg/ml α-casein
• Imaging Buffer (IB): MB-KCl buffer supplemented with 1.2 μM glucose oxidase, 0.2 μM catalase, 4.2 μM alpha-casein, 17 mM glucose, and 20 mM DTT. (Tip: gluocose oxidase and catalase must be prepared fresh from powder; alpha-casein can be stored in 10 mg/ml aliquotes at −80 °C for several months; glucose can be stored at 250 mg/ml at 4 °C for several weeks. Owing to deterioration of the enzymes, IB must be prepared fresh every ˜ 5 h)
• Imaging Buffer with calmodulin (IB-C): IB buffer supplemented with 2 μM CaM
• Reaction Mix (RM): IB supplemented with 0.1 mg/ml creatine phosphokinase, 5 mM creatine phosphate, 2 μM CaM and ATP at the desired concentration (Tip: RM must be prepared fresh for every assay)
• High Salt (HIS) Buffer 2x (pH 7.5): 1 M KCl, 40 mM KPi, 2 mM EGTA, 4 mM MgCl_2_ (Tip: filter sterilize and store it at 4 °C for several weeks)
• NEM-myosin II (20 mg/ml) in HIS 2x (see protocol in Batters and Veigel [14]) (Tip: store NEM-myosin II on ice and use it for 2–3 days)
• μl of previously prepared bio-HMM-5B conjugated to streptavidinated QDs 655 (QD-HMM-5B) 40 nM (Section 5.1)
• Rhodamine-phalloidin F-actin (Section 2)

Methods:

1 Prepare a flow cell as described in Section 3 and leave it on ice (all the following operations, except observations under the microscope, have to be carried out on ice)
2 Prepare all buffers and leave them on ice
3 Flux NEM-Myosin II 2 mg/ml in HIS 2x and incubate for exactly 1 min
4 Wash with 3 volumes of MBα
5 Flux 23 nM rhodamine phalloidin F-Actin (in MB-KCl supplemented with 10 mM DTT) and wait for 2 min.
6 Wash with 4 volumes of IB
7 Dilute 5 μl of QD-HMM-5B in 95 μl IB-C to get 2 nM QD-HMM-5B. (Tip: this dilution can be used for 4–5 hours, after that a new QD-HMM-5B conjugation (section 5.1) must be done)
8 (Optional) Dilute QD-HMM-5B prepared in step 7 to 0.1 nM in IB-C and flux it in the chamber and check under the microscope that a reasonable number of QDs are attached on actin filaments.
9 Dilute QD-HMM-5B prepared in step 7 to 0.1 nM in RM at the desired ATP concentration and flux it in the chamber.
10 (Optional) Close carefully the chamber with silicone grease. Alternatively, leave the flow cell open to change the QD-HMM-5B and ATP concentration on the same sample.
11 Run under the microscope for watching myosins moving along actin filaments!

### Image acquisition

Images are acquired on a modified Nikon ECLIPSE TE300 inverted fluorescence microscope [4] equipped with a 532 nm laser for rhodamine excitation and a 488 nm laser for QD excitation. Images were acquired in total internal reflection configuration, through a Nikon Plan Apo TIRF, 1.45 NA, 60X, oil immersion objective. 91 nm pixel size images were obtained by projecting the fluorescence image onto an iXon 3 EMCCD camera, after an additional 3X magnification through an achromatic doublet telescope.

**Fig. 5:**
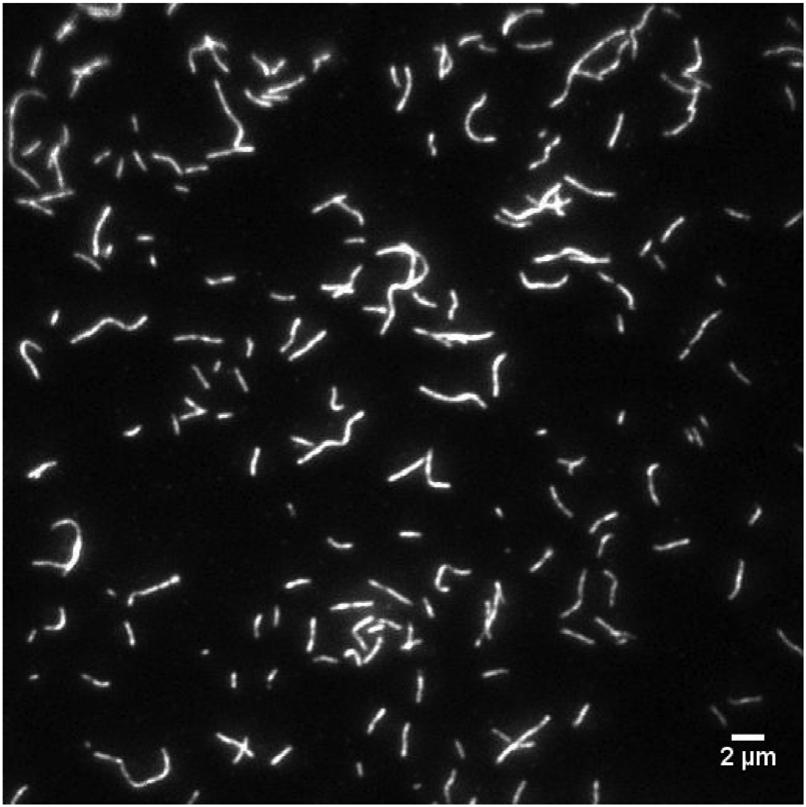
The gliding assay. Image of fluorescent actin filaments bound to myosin in the in vitro gliding assay.

For the gliding assay: F-actin Exc/Em. 532/580 nm. Laser power on the sample was about 3 mW, 200 ms exposure time and 200 EM gain are usually fine (Fig. 5).

**Fig. 6:**
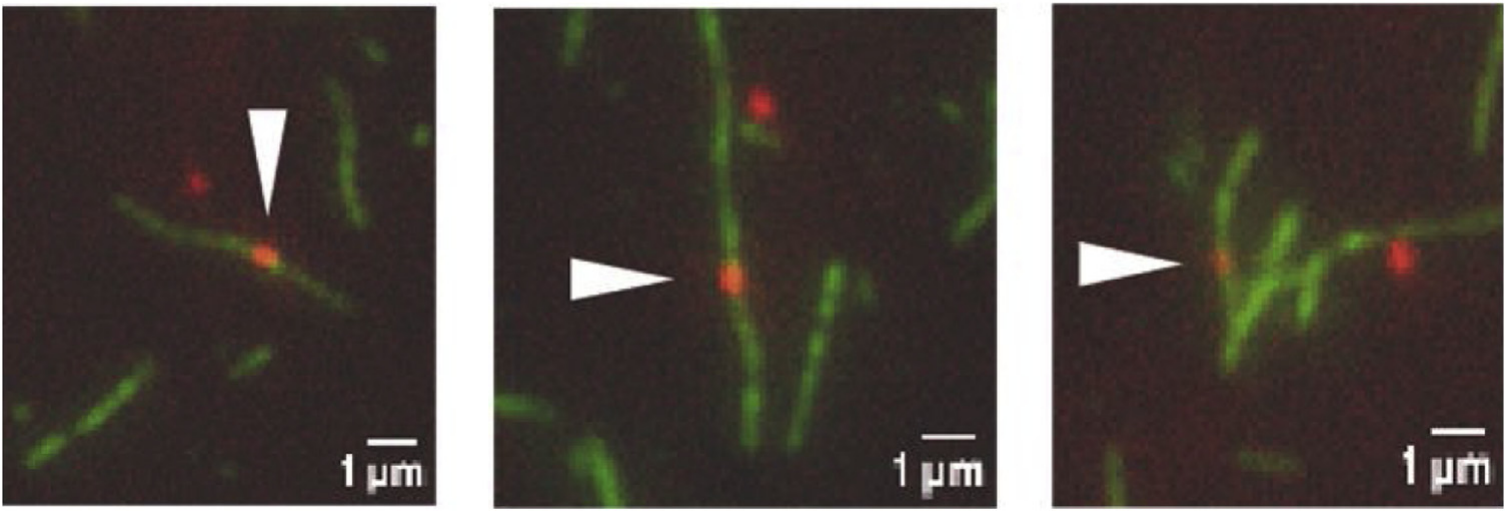
The single molecule motility assay. Images of single QDs (red) moving along fluorescent actin filaments (green) bound to the coverslip in the single molecule motility assay. C.

For the single molecule motility assay: F-actin Exc/Em. 532/580 nm, laser power on the sample was about 3 mW; QD-HMM-5B Exc/Em. 488/655 nm, laser power on the sample was about 3 mW. Exposure time is set according to ATP concentration and consequent velocity between 50 and 100 ms. EM gain was 300 (Fig. 6).

### Data analysis

Gliding assay: single filaments characterized by a travelled distance >1 μm were tracked over the frames with the ImageJ Plugin “Multitracker”. The filament velocity was calculated frame by frame and the average velocity with its standard error was associated to each filament. The final average velocity was calculated by weighted average with associated standard error. By this analysis we obtained an average gliding velocity of 163 ± 3 nm/s at 1 mM ATP (N = 20 filaments).

Single molecule motility assay: single QDs were localized frame by frame with nanometer precision by a custom-made software named “PROOF” that is freely available and widely described elsewhere [4]. Localization accuracy in single molecule motility experiments was around 4 nm (100 ms integration time, 300 EM gain, 3 mW laser power on the sample), calculated as standard deviation of immobile QDs on actin filaments over 20 frames, possibly limited by the compliance of the NEM-myosin attachment to the coverslip. Myosin trajectories were derived from x,y coordinates of QDs moving along actin filaments for more than ten frames. Analysis of step size, run length, and velocity based on myosin-5B trajectories under variable ATP concentration can be found in Gardini et al. [7].
